# Past rewards capture spatial attention and action choices

**DOI:** 10.1007/s00221-013-3654-6

**Published:** 2013-08-14

**Authors:** E. Camara, S. Manohar, M. Husain

**Affiliations:** 1 UCL Institute of Cognitive Neuroscience, 17 Queen Square, London, WC1N 3AR UK; 2 UCL Institute of Neurology, Queen Square, London, WC1N 3BG UK

**Keywords:** Oculomotor capture, Distractibility, Eye movements, Reward

## Abstract

The desire to increase rewards and minimize punishing events is a powerful driver in behaviour. Here, we assess how the value of a location affects subsequent deployment of goal-directed attention as well as involuntary capture of attention on a trial-to-trial basis. By tracking eye position, we investigated whether the ability of an irrelevant, salient visual stimulus to capture gaze (stimulus-driven attention) is modulated by that location’s previous value. We found that distractors draw attention to them significantly more if they appear at a location previously associated with a reward, even when gazing towards them now leads to punishments. Within the same experiment, it was possible to demonstrate that a location associated with a reward can also bias subsequent goal-directed attention (indexed by action choices) towards it. Moreover, individuals who were vulnerable to being distracted by previous reward history, as indexed by oculomotor capture, were also more likely to direct their actions to those locations when they had a free choice. Even when the number of initial responses was made to be rewarded and punished stimuli were equalized, the effects of previous reward history on both distractibility and action choices remained. Finally, a covert attention task requiring button-press responses rather than overt gaze shifts demonstrated the same pattern of findings. Thus, past rewards can act to modulate both subsequent stimulus-driven as well as goal-directed attention. These findings reveal that there can be surprising short-term costs of using reward cues to regulate behaviour. They show that current valence information, if maintained inappropriately, can have negative subsequent effects, with attention and action choices being vulnerable to capture and bias, mechanisms that are of potential importance in understanding distractibility and abnormal action choices.

## Introduction

There is growing evidence for the effects of reward on the deployment of visual attention (for recent reviews, see Anderson 2013; Chelazzi et al. 2013). That selective attention plays a role in guiding behavioural choices and that such choices are also affected by rewards and punishments have been established for some time. However, whether attention is directly modulated by reward has, perhaps surprisingly, not been explored until relatively recently (see Chelazzi et al. 2013). Investigations that have manipulated rewards associated with particular target features have shown that these can alter behaviour (indexed by reaction times) in, for example, visual search and negative priming experiments (Della Libera and Chelazzi 2006; Kiss et al. 2009).

The results of some studies have suggested that reward-associated features directly attract attention by bottom-up, stimulus-driven mechanisms (Hickey et al. 2010; Anderson et al. 2011; Theuwees and Belopolsky 2012). That is, a stimulus that is associated with reward may become more attractive by modification of its visual salient properties. For example, using a visual search task, Hickey et al. (2010) showed that participants responded faster on those trials where a target had the same colour as a previously rewarded one. More importantly, when a distractor was presented with the same previously rewarded colour, high-attentional capture occurred with slowing of responses compared to distractors of the same colour as previously low-rewarded targets. Additionally, when ERPs were analysed, an increase in mean amplitude of the lateral P1 component (typically localized to early visual areas and associated with increased salience) was observed following high rewards. From these results, Hickey et al. (2010) concluded that the reward-related selection mechanisms might intrinsically change properties of human perception, accounting for automatic, *value*-*driven attentional capture*.

In real-world situations, such attentional capture by rewarded stimuli might be extremely important for some behavioural decisions, allowing rapid orienting of attention to unexpected—but potentially valuable—information. Thus, learning about features in the visual environment that are predictive of high-reward outcome might be crucial for successful behaviour. Indeed, reward effects on attentional capture through associative learning have now been reported across a range of paradigms, including negative priming, visual search and oculomotor capture (Della Libera and Chelazzi 2006, 2009; Anderson et al. 2011; Theuwees and Belopolsky 2012). In these experiments, participants undergo a learning phase where they are exposed to stimuli associated with different rewards. Thereafter, in a subsequent phase, there are clear effects of previously rewarded features in capturing attention, indexed either by reaction time slowing or capture of gaze. Along the same lines, electrophysiological recordings in rhesus monkeys have also shown that visual processing of objects predicting positive outcomes is enhanced compared with those associated with negative outcomes (Platt and Glimcher 1999; Ikeda and Hikosaka 2007, Peck et al. 2009). 

Although value-driven attentional capture might be a useful adaptive mechanism for an organism, might it also have detrimental effects? In some ways, previous studies have shown that this is possible, e.g. singleton distractors with the same colour as previously rewarded targets can slow down visual search (Hickey et al. 2010; Anderson et al. 2011). However, what is unclear from these experiments is how a previously rewarded attribute might compete with a new feature that is now highly rewarded. Using an oculomotor capture paradigm, it has now been demonstrated that saccadic curvature towards and away from a distractor is modulated by previous reward history associated with a distractor, even when participants are instructed to ignore the distractor (Hickey and van Zoest 2012).

But is it possible that attention would still be captured by a previously rewarded feature even if it is now associated with a *penalty*—and despite the fact that a new feature is available with high-reward delivery associated with it? Furthermore, does a rewarded attribute that captures attention in an involuntary way also affects subsequent goal-directed action choices when participants are given a free choice about where to deploy attention? The oculomotor capture paradigm (Theeuwes et al. 1999) provides one way to investigate these issues, allowing us to examine directly whether previously valued locations influence where people choose to look next.

Here, we examine these issues using a design that allowed us to assess how the value associated with a location subsequently affects involuntary capture of attention as well as the deployment of goal-directed attention. We first used an oculomotor capture paradigm, to investigate whether the ability of an irrelevant distractor to attract gaze is affected by that location’s previous value, even when gazing towards the distractor is associated with a penalty. In these experiments, there was no long-term associative learning between a reward and location because target and distractor locations changed on each trial. Crucially, we modified the task to investigate if such capture is modulated by whether the distractor occupies a location that on the previous trial had been rewarded or penalized.

In addition, within the same experiment, we assessed whether such valence information also affects where participants direct their gaze if they are allowed to choose freely where to look. Finally, we examined whether covert attention is modulated in a similar way to overt gaze shifts, using a manual response paradigm. Across all three experiments, our results show that past rewards can act powerfully—and sometimes detrimentally—to affect the deployment of both stimulus-driven and goal-directed attention, even when the value of locations alters on a trial-to-trial basis.

## Materials

### Participants

In the eye-tracking experiments, fourteen participants (9 women, aged 20–35, mean 26.6, SD 3.8) took part in Experiment 1, eleven in Experiment 2 (10 women; aged 19–29, mean 24.1, SD 3.3) and five participated in both. In the manual button-press task (Experiment 3), seventeen new participants took part (11 were women, age 19–37, mean age 26.4 (SD 4.4)). All gave written informed consent. All procedures were approved by the local ethics committee. Participants were right-handed, with no history of neurological or psychiatric episodes and had normal visual acuity.

### Apparatus

Stimuli were displayed on a black background on a 21″ CRT monitor (1,024 × 768 pixels, refresh rate 150 Hz) at a viewing distance of 60 cm. Eye position was monitored at 1,000 Hz (Eyelink 1000; SR Research) and parsed online to give feedback. For the eye-movement experiments, saccade initiation was signalled when eye velocity exceeded 30° s^−1^, acceleration exceeded 8,000°^−2^ and amplitude of gaze shift was a minimum of 0.15°. The endpoints of saccades were determined with respect to whether they fell within the circumference of a stimulus presented on the screen (coin stimulus or target/distractor).

### Experiment 1

**Fig. 1:**
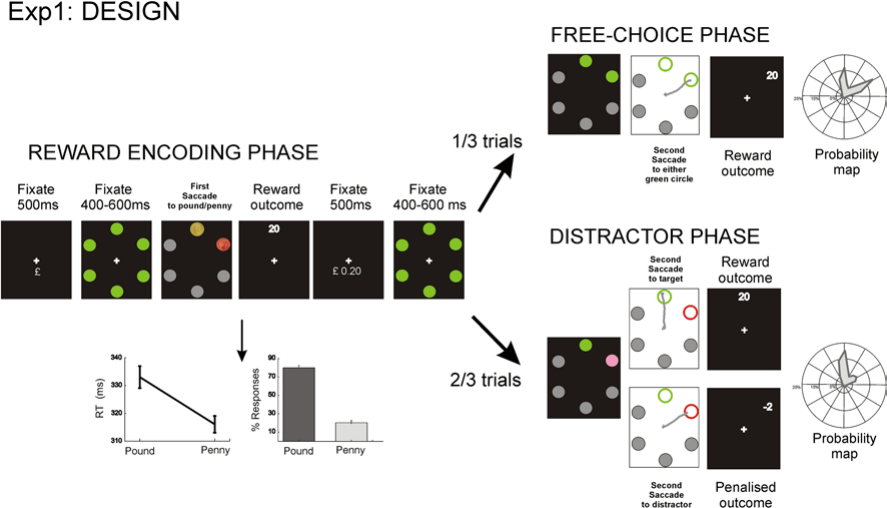
Experiment 1. A reward-encoding phase (*Phase 1*) was followed either by a free-choice or distractor phase (*Phase 2*). SRTs and percentage of responses to the pound or penny are shown in the first phase. An example saccade in the free-choice and distractor phase is depicted, together with group-average angular polar histograms distributions showing probability of gaze shifts to the two discs (both *green* in free choice; *one* *pink* distractor and *one green* target in the distractor phase)

The aim was to determine whether reward information encoded in the first phase of each trial altered goal-directed and stimulus-driven behaviour in the second phase. Each trial started with a reward-encoding phase followed by a probe phase. Importantly, these were not linked in any way, apart from the fact that the latter followed the former. Thus, behavioural response in the second phase was crucially not contingent upon the response made in the first part of the experiment (Fig. 1 illustrates the paradigm).

In the first—reward-encoding—phase, monetary cues were used to associate reward information with spatial locations. In the subsequent probe phase, the influence of reward associations established at the reward-encoding phase was probed in two different ways:

- ‘Free-choice’ condition, where participants chose where they would look between two possible alternatives and
- ‘Distractor’ condition, where subjects were shown the target to look at, but this was accompanied by a distractor.

The free-choice condition allowed us to assess the influence of reward or punishment in the encoding phase on subsequent, independent goal-directed behaviour. The distractor condition permitted us to examine the affect of previous reward or penalty on stimulus-driven capture of behaviour. The delay between phase 1 and phase 2 of the experiment was 2 s.

In the reward-encoding phase (Phase 1), six equidistant 3° green circles were presented at 11° eccentricity on an imaginary circle centred on the fixation point (Fig. 1). Next, four of the green circles changed to grey and two adjacent circles turned into a pound and a penny coin (of equal visual luminance). Monetary cues were used to associate reward information with spatial locations. Participants were instructed to saccade from the central fixation point towards the pound as fast as possible. When they looked at the pound, they were rewarded; when they went to the penny, they were penalized by a fixed amount (reward and penalty details below).

Then, the six green circles were re-presented, and participants fixated centrally until the phase 2 commenced. If this phase was a free-choice condition (one-third of phase 2 trials), four of the green circles changed to grey, but the two circles where coins had previously been presented remained green. Participants were required to make a saccade of their choice to either of the two green circles (targets) and were rewarded on the basis of their reaction time. This formed our measure of goal-directed behaviour, allowing us to determine whether reward/penalty in the previous encoding phase affected subsequent choice.

By contrast, our measure of stimulus-driven behaviour was indexed by the influence of distractors in capturing gaze. In the distractor condition (two-thirds of phase 2 trials), four of the green circles changed to grey, but a target circle (green) and a distractor circle (pink) were now presented at the locations previously occupied by the coins. Participants had to saccade to the green target, but on occasions their gaze was captured by the salient pink distractor—‘oculomotor capture’ (Theeuwes et al. 1999). If participants made a saccade to the green target, they were rewarded on the basis of their reaction time; otherwise, they were penalized.

To avoid unwanted second-order reward history effects, new pairs of coin positions were generated from trial-to-trial. Thus, pairs of coins never appeared at any previous location in consecutive trials. Moreover, different pairs of coins and distractor-target locations were all counterbalanced in order, resulting in 12 configurations for the free-choice condition and 24 for the distractor-capture condition. Each participant completed eight blocks of 36 trials (12 free choice; 24 distractor capture), all pseudo-randomly presented. Trials with SRT <100 ms or >1,000 ms were rejected from analysis.

### Experiment 2

**Fig. 2:**
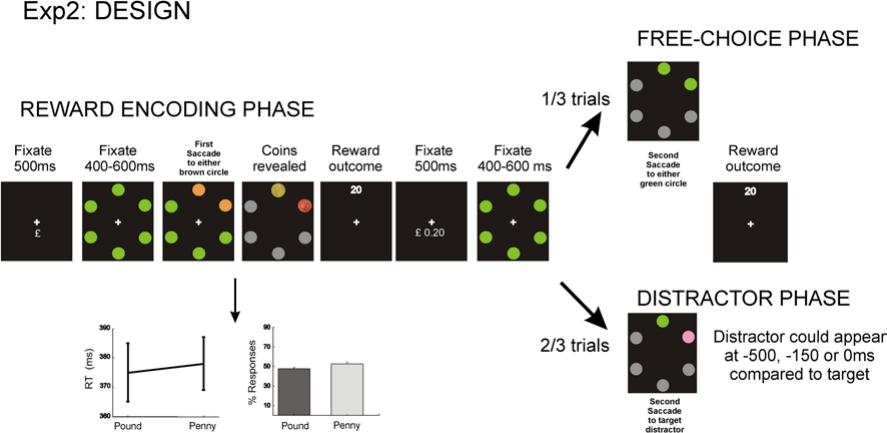
Experiment 2. In the reward-encoding phase, the identity of coins was not revealed until the saccade was completed, so percentage of responses to rewarded versus penalized locations was not significantly different. Proportion of saccades and SRTs are plotted below. *Error bars* indicate SEM

Participants performed a very similar task, with a reward-encoding and probe phase (one-third trials free choice; two-thirds distractor capture). But in this experiment, coin identity was not revealed until the saccade was completed in the reward-encoding phase (Fig. 2). Thus, initially, participants chose freely which one of two brown circles they would saccade to, after which either a pound or a penny was revealed (selected randomly) at that location. Positive or negative feedback was provided based on the cue value (pound or penny) and reaction time (details below). In this way, the number of saccades generated towards rewarded and penalized locations was equalized in this experiment.

Moreover, in the distractor-capture condition, in order to manipulate stimulus salience of the distractor, we varied the time interval between presentation of distractor and target. Stimulus onset asynchrony (SOA) was either 500, 150 or 0 ms. Thus, the distractor could appear before the target in two-thirds of trials and simultaneously on one-third. Each participant completed 10 blocks, comprising 12 free-response trials and 24 distractor-capture trials (8 trials for each SOA) in each block, all of them pseudo-randomly presented.

### Experiment 3

Finally, we ran a covert attention version of the paradigm using manual responses instead of eye movements. This experiment was very similar to the previous experiments, but instead of making saccades, participants used button-press responses to indicate their choice. First, analogous to Experiment 2, in the encoding phase, coin identity was not revealed until participants’ response (in this case button-press) was completed, in order to keep number of responses to rewarded and penalized locations equal. In the second (probe) phase, as in the Experiment 1, free choice and distractor trials were randomly presented. In this case, half the trials were free choice (with participants choosing freely between the two identical green circle targets that appeared at the locations where the coins were presented), and half of the trials corresponded to the distractor condition (with a target green circle and a pink distractor circle simultaneously presented at the locations previously occupied by the coins).

Participants were asked to fixate centrally in every trial. They were instructed to make their manual responses regarding their choice of target circle as soon as targets, or target and distractor, appeared. Pound and penny, as well as target and distractor stimuli were easily distinguishable from fixation. Participants used two keyboard keys (‘g’ and ‘h’), corresponding to responses to the leftmost or rightmost circle. They responded with two hands. Each participant completed five blocks, comprising 24 free-response trials and 24 distractor-capture trials, in each block. Trials with manual responses <150 ms were classified as anticipatory responses and discarded from analysis.

### Reward function and penalties

Reward was calculated based on saccade reaction time (SRT) by an exponential decaying discounting function in the form: $R = {\text{Ae}}^{{ - {\it{t}} - {{{\it{t}}_{{\min }} } \mathord{\left/ {\vphantom {{{\text{t}}_{{\min }} } \tau }} \right. \kern-\nulldelimiterspace} \tau }}},$ truncated to the nearest integer, where *A* = 20, *t*_min_ = 335 ms, *τ* = 35 ms and *t* represents the SRT. For SRT faster than *t*_min,_ the maximum amount (20) was obtained. For the manual response experiment, reward was also calculated by an exponential decaying discounting function based on manual reaction time (RT), in the form of: $R = {\text{Ae}}^{{ - {\it{t}} - {{{\it{t}}_{{\min }} } \mathord{\left/ {\vphantom {{{\text{t}}_{{\min }} } \tau }} \right. \kern-\nulldelimiterspace} \tau }}},$ truncated to the nearest integer, where *A* = 20, *t*_min_ = 350 ms, *τ* = 100 ms and *t* represents the manual reaction time. Additionally, for both the saccadic response and the manual response experiments, erroneous responses were penalized by a fix amount (−2 pence). Neither reward nor penalty (0) was delivered for SRT/RTs >500 ms for responses to either the pound (in reward-encoding phase) or green target (in probe phase), to encourage participants to respond quickly. Participants were informed that (0) non-reward values corresponded to correct, but too slow responses.

The amount won or lost was displayed at the selected target circle for 1 s. Additionally, four different sounds were played when the reward-related feedback was displayed [High reward (HR): 20 pence/jack-pot sound; Low-reward (LR): 19–1 pence/drop coin sound; Non-Reward (NR): 0 pence/beep; Punishment (P): −2 pence/error beep]. Participants were told they might win a maximum of 15 pounds. They were instructed to maximize reward, by increasing speed of responses in free-choice trials and avoiding capture by the distractor in distractor trials. At the end of each block, participants were told the total amount they had earned in that block. At the end of the experiment, all participants were given 15 pounds.

## Results

### Experiment 1

#### Reward affects gaze in encoding phase

In the reward-encoding phase, participants were presented with pairs of coins (pound and penny) at adjacent locations, with associated feedback on performance (reward or punishment). As expected, they chose the valued location (pound) far more often than the penny [Pound vs. Penny, mean (SEM): 79.9 (2.4) vs. 20.1 % (2.4), *t* (13) = 12.2, *P* < 0.0001]. Importantly, SRTs were significantly longer for saccades to the rewarded location compared to the penalized one, [Pound vs. Penny: 333 (4) vs. 316 ms (3), *t* (13) = 6.12, *P* < 0.001; see Fig. 1].

#### Past reward modulates free-choice responses

**Fig. 3:**
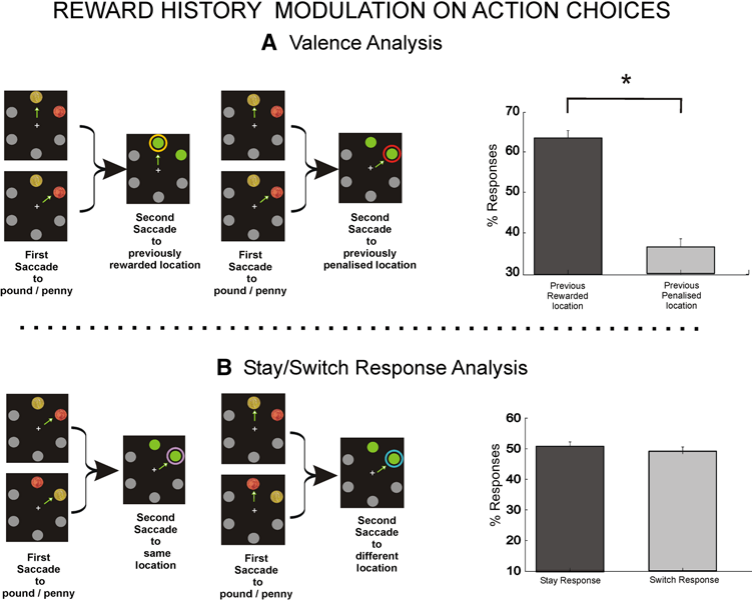
Reward history modulates action choices. The valence analysis (**a**) shows more saccades in the free-choice phase went to previously rewarded locations. In the stay/switch motor response analysis (**b**), there was no difference between saccades made to the same or different location from phase 1, regardless of whether previous saccades had gone to a rewarded or a penalized location. *Error bars* show SEM

In the subsequent probe phase one-third of trials was free choice, with participants choosing freely between the two identical green circle targets that appeared at the location where the coins had been. Significantly, more saccades were directed to the previously rewarded location [Fig. 3a; rewarded: 63.5 % (2) vs. penalized: 36.5 % (2), *t* (13) = 6.8, *P* < 0.0001]. No differences were observed in SRTs [rewarded: 419 ms (4.5) vs. penalized: 410 ms (6.1)]. Importantly, since both target saccade locations were identical in this phase, and therefore did not differ in visual salience, the result can be explained only by the maintenance of history-valence contingencies. Moreover, the valence selectivity seems unlikely to be due to the establishment of a strategic set, given those are typically modulated by reaction time differences (Prinzmetal et al. 2005).

However, since previously rewarded locations were chosen more frequently in the first phase of the experiment, this analysis does not exclude the possibility that the result was simply due to ‘motor perseveration’, i.e. a tendency simply to repeat the saccade made in the encoding phase. We therefore analysed the difference between saccades made to same versus different location in the two phases, *regardless* of whether that location corresponded previously to a penny or pound (Fig. 3b). Crucially, there were no significant effects when such global response repetition and global response switch responses (irrespective of previous rewards or penalties) were compared, neither for percentage of saccades [stay vs. switch: 50.8 % (1.5) vs. 49.2 % (1.5)] nor for SRTs [stay vs. switch: 420 ms (5.3) vs. 405 ms (4.8)]. Thus, goal-directed action choices show a preference for a previously rewarded location that cannot be explained by simple response perseveration. In other words, going back to a location here was driven by past reward outcome.

#### Past rewards capture stimulus-driven behaviour

**Fig. 4:**
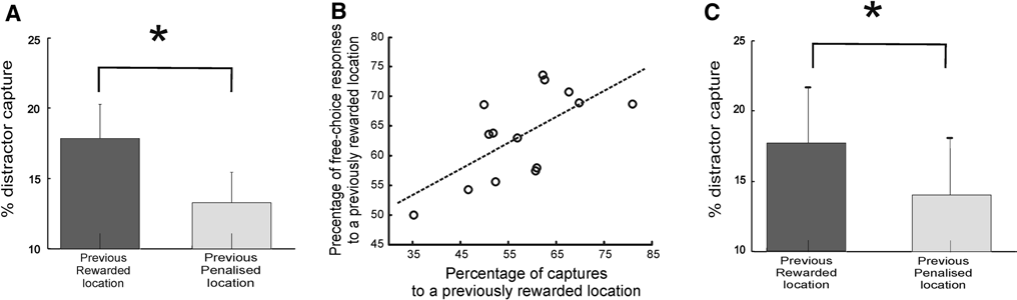
Reward history captures attention. Mean percentage of saccades in Experiment 1 (**a**) and Experiment 2 (**c**) to previously rewarded locations compared to previously penalized locations, in the distractor-capture phase. Distractors attracted gaze to them significantly more if they appeared at a location that had previously been rewarded, even when gazing towards them now led to punishments. *Error bars* indicate SEM. **b** There was a significant positive correlation between the relative percentage of *free*-*choice* responses to previously rewarded locations and the relative percentage of *capture* responses to previously rewarded locations (Experiment 1)

In the distractor phase, we examined whether gaze capture by the salient distractor was modulated by the reward history associated with its location. Visually salient distractors captured gaze on 31.2 % (4.2) of the trials [cf. target 68.8 % (4.2)]. Note that this occurred despite the fact that saccades to distractors led to a penalty. Such ‘capture’ saccades had significantly shorter SRTs than those to targets [distractor vs. target: 326 (4) vs. 368 ms (10), *t* (13) = 5.1, *P* < 0.001]. Crucially, distractors presented at a previously rewarded position in the trial significantly increased capture compared to distractors presented at a previously penalized location [17.9 % (2.3) vs. 13.3 % (2.1), *t* (13) = 3.35, *P* < 0.005; Fig. 4a]. Thus, reward history affected stimulus-driven behaviour, even when visual salience of the distractor was not different.

Previous studies have reported that such capture is more likely on trials with shorter SRTs (Theeuwes et al. 1999). Importantly, in our experiment, there were no significant differences when SRTs were compared for responses made to previously *rewarded* versus previously *penalized* locations [324 ms (4) vs. 330 ms (5)].

Does vulnerability to distraction by previous reward correlate with free-choice behaviour? Importantly, there was a significant correlation between the relative percentage of *free*-*choice* responses to previously rewarded locations and relative percentage of *capture* responses to previously rewarded locations (*r* = 0.66, *P* = 0.001), even though free-choice trials were on completely different trials to distractor ones (Fig. 4b). Thus, individuals who were more vulnerable to stimulus-driven capture by reward were also biased in making more action choices to previously rewarded locations.

Although in principle reward and punishment encoding is separable in the first phase of our experiment, rewarded targets were chosen significantly more frequently in this phase. Moreover, penalized responses might induce a differential slowing effect, similar to the automatic ‘cognitive control’ triggered after an error (Rabbitt 1966; Emeric et al. 2007; Klein et al. 2007), potentially confounding interpretation. We therefore ran a second experiment which equalized rewarded and penalized responses in the encoding phase.

### Experiment 2

#### Past rewards modulate goal-directed and stimulus-driven behaviour

This experiment was very similar to Experiment 1, but in the first phase, participants made saccades freely to one of two adjacent brown circles. The coin values—assigned randomly to these locations—and their associated performance feedback were not revealed *until the saccade was completed*. Thus, participants’ responses to rewarded and penalized locations were not significantly different.

Consistent with the previous results, in the free-choice condition (phase 2 of experiment), reward history modulated action choices. Thus, significantly more saccades went to previously rewarded locations [rewarded vs. penalized: 59.5 % (2.5) vs. 40.5 % (2.5), *t* (10) = 3.9, *P* < 0.003]. But SRTs did not differ [rewarded vs. penalized: 368 ms (2.7) vs. 371 ms (4)]. In the distractor condition, when salient distractors were simultaneously presented with the target, distractors captured gaze significantly more often if they were located at a previously rewarded location compared to a penalized one [rewarded: 17.6 % (3.9) vs. penalized: 13.9 % (4.1), *t* (10) = 3.01, *P* < 0.013; Fig. 4c].

#### Dynamics of oculomotor capture and reward modulation

To investigate how visual salience and previous reward information interact, we increased the visual salience of the distractor by altering SOA between distractor and target onset (0, 150 or 500 ms). Percentages for oculomotor capture were analysed in a 3 × 2 repeated measures ANOVA, testing the effects of SOA (0, 150, 500 ms) and reward history (previous rewarded location relative to penalized).

First, as expected, delaying target presentation led to significantly increased oculomotor capture [main effect SOA, 0 ms 31.5 % (8); −150 ms 36.9 % (7.7); −500 ms 38.8 % (7.5); *F*(2,20) = 7.8 *P* < 0.003]. With regard to the effects of reward history on capture, a main effect of previously rewarded location was observed, across SOAs [rewarded: 19.3 % (3.8), penalized: 16.5 % (4), *F*(1,10) = 7.7, *P* < 0.019]. Although significant interaction effects were not observed, in order to replicate previously reported reward history effects, we investigated valence selectivity when the target and the distractor occurred simultaneously. Importantly, a significant effect of reward history on capture was present in this condition. [0 ms, rewarded: 17.6 % (4) vs. penalized: 13.9 % (4.1), *t* (10) = 3.014, *P* < 0.013]. Further exploratory analysis showed that at longer SOAs, there was no such effect [−150 ms, rewarded: 20.2 % (3.9) vs. penalized: 16.7 % (4.1); −500 ms, rewarded: 20.1 % (3.8) vs. penalized: 18.7 % (3.9)]. Overall, these results indicate that a stimulus-driven mechanism contributes significantly to attention guidance, and suggest that reward history also plays at role, although only when visual salience-driven influences are relatively weaker.

### Experiment 3

#### Past rewards modulate goal-directed and stimulus-driven manual responses

Consistent with the saccade results, in both the free-choice and the distractor condition, reward history modulated manual response choices, while no significant differences were observed in corresponding reaction times. In particular, in the free-choice condition, participants pressed significantly more often to previously rewarded locations [rewarded vs. penalized: 73.8 % (2.7) vs. 26.2 % (2.7), *t* (16) = 9.1, *P* < 0.0001]. But RTs did not differ [rewarded vs. penalized: 476 ms (29) vs. 468 ms (27)].

In the distractor condition, in which salient distractors were simultaneously presented with the target, participants made very few errors in the distractor condition. Nevertheless, participants committed significantly more errors when the distractors were located at a previously rewarded location compared to a penalized one [rewarded: 8.8 % (2.02) vs. penalized: 5.1 (1.3) %, *t* (16) = 2.4, *P* < 0.03]. Additionally, no significant differences were observed when the RT errors were compared. Very few trials were excluded from analysis [1.1 % (0.7)] because they were anticipatory (<150 ms). These findings strongly support the previous results, demonstrating that the previous value of a location subsequently affects deployment of goal-directed attention as well as involuntary capture of attention on a covert attention task.

## Discussion

The findings presented here show that valence information in healthy people is dynamically maintained from trial-to-trial, drawing attention towards previously rewarded locations, even when this interferes with the task at hand. Such effects were present for both stimulus-driven and goal-directed deployment of attention. Thus, in situations without any particular attentional priority (no difference in visual salience or goal relevance of potential targets) and where observers freely choose where to attend to, rapid saccadic or button-press choices were also preferentially attracted to previously rewarded locations. These findings demonstrate that a location associated with a positive outcome can bias goal-directed attention towards it, even when rewarded locations are changing on a trial-to-trial basis, so that overall no particular location is reinforced over time as having a high value.

Previous studies have also demonstrated that features associated with rewards in the past can interfere with current deployment of selective attention. For example, singleton distractors with the same colour as previously rewarded targets slow visual search (Hickey et al. 2010; Anderson et al. 2011), while saccadic curvature towards and away from a distractor is modulated by previous reward history associated with that distractor’s colour (Hickey and van Zoest 2012). However, to the best of our knowledge, it has not been convincingly demonstrated that attention can be captured by a previously rewarded feature when it is now associated with a *penalty*—and despite the fact that a new feature is available with high-reward delivery associated with it; That is, in a context where an old highly reward-feature attribute competes directly with a new one, and there is a clear penalty associated with directing attention to the old feature. The findings presented here show that exactly this can occur, with either overt or covert attention shifts.

Some investigators have proposed that reward associated with a feature might alter its intrinsic visual salience (Hickey et al. 2010; Theuwees and Belopolsky 2012). The results presented here would certainly be consistent with this view, since we found that differences in reward history associated with a location modulate oculomotor capture, a stimulus-driven mechanism that appears to be guided by visual salience (see also Hickey and van Zoest 2012). Therefore, one interpretation of our results is that valence information might potentially modify the salience of a stimulus and disrupt ongoing goal-directed behaviour. But the paradigm used here allowed us to examine whether a rewarded location that captures attention in an involuntary way also affects subsequent goal-directed action choices when participants are given a free choice about where to deploy attention. The results demonstrate that the effects of reward on free choices are indeed similar, demonstrating that valence information can be maintained from previous trials, even inappropriately, also to bias goal-directed attention.

Previous trial effects might originate from different potential mechanisms. One possibility is that reward information is active in working memory (WM) from trial-to-trial, affecting subsequent action choices by guiding attention. Recent studies have indicated a robust connection between WM and selective attention, in such a way that active memory representations determine which perceptual objects are selected (see review by Olivers et al. 2011). It has been argued that normally only one item acts as a template to guide attention, pushing aside any other (‘accessory’) items in WM (Olivers et al. 2011). But, of course, if accessory items are not inhibited they might intrude on current behaviour. Thus, a location that had previously been associated with a reward might be inappropriately maintained in WM to affect subsequent behaviour. One recent study has demonstrated that loading WM (using an *N*-back letter identity task) can also increase oculomotor capture (Van der Stigchel 2010), but note that our task did not require participants to maintain previous trial information, yet nevertheless past reward outcomes intruded on current performance.

A second possibility to consider is the role of priming. Pop-out priming in visual search is in many ways related to the effects of reward on attention: a previous salient target subsequently captures attention faster, just as a highly rewarded target does. Brascamp et al. (2011) used a novel procedure, analogous to the one that we have applied but in which pop-out trials were intermixed with subsequent free-choice trials. Importantly, when participants were instructed to freely choose which item to attend to, they strongly drove attention to the preceding pop-out target. Conversely, free-choice trials also speeded subsequent pop-out search responses. These findings suggest that target selection is based on relative salience and priorities, altered by selection history. Thus, reward information might be represented as a dynamically evolving peak of activity in a map-like representation of spatial priority of the visual scene, where physically salient items compete with previous valence history. A previously rewarded location might modify the relative representation of a location by differentially changing the activity in neurons (e.g. in frontal eye fields or superior colliculus for eye movements), allowing responses to be initiated faster when the target next appears at that location (Fecteau and Muñoz 2003).

Finally, an important aspect of the results of our study is that maintenance of past valence information can be potentially maladaptive. By contrast, some previous studies have emphasized its functional utility in guiding visual search to maximize reward (Navalpakkam et al. 2010; Kristjánsson et al. 2010). Although it is easy to see how useful it is for attention to be directed to potentially highly rewarding features in the visual scene, valence information associated with singleton distractors can also have negative consequences, slowing down responses to targets (Hickey et al. 2010; Anderson et al. 2011). In our experiment, individuals who were more vulnerable to attentional capture by previous reward history were also more likely to direct their actions to those locations even when they had a free choice. These findings might be important in understanding distractibility and abnormal action choices.

In our paradigm, distractibility can be indexed by the ability of an irrelevant visually salient stimulus to capture gaze, or for a previously rewarded location to draw attention to it inappropriately. Such behaviour raises questions about distraction and perseveration in pathological states, e.g. following frontal injury. Despite being widely recognized in focal as well as degenerative brain disorders, very little is understood about the mechanisms underlying distractibility and perseveration (Ridley 1994; Hotz and Helm-Estabrooks 1995). Traditionally, such observations in the ‘frontal lobe syndrome’ (Luria 1965; Woods and Knight 1986) have tended to be viewed in terms of poor response inhibition (Aron 2007). But, pathologically inappropriate maintenance of features or locations in WM might be another important mechanism whereby such maladaptive behaviour might arise. Thus, in our experiments in healthy people, goal-directed action choices demonstrated a preference for a previously rewarded location: going back to a location was driven by past reward outcome.

In pathological states, such as in frontal dysfunction, such effects might be further heightened if a location is inappropriately overvalued. Frontal regions including orbitofrontal and ventromedial prefrontal cortex are thought to play a key role in encoding stimulus value (Kringelbach and Rolls 2004; O’Doherty 2004; Wallis 2007). How brain representations are dynamically updated so that previous valence information is maintained when appropriate and destroyed when unhelpful is a potentially important avenue of research for both behavioural and physiological studies, in health and disease.
